# Defining Trimester-Specific Reference Intervals for Thyroid Hormones: Insights from a Bulgarian Monocenter Study

**DOI:** 10.3390/medicina60050801

**Published:** 2024-05-13

**Authors:** Vesselina Yanachkova, Radiana Staynova, Emilia Krassimirova Naseva

**Affiliations:** 1 Department of Endocrinology, Specialized Hospital for Active Treatment of Obstetrics and Gynecology “Dr Shterev”, 1330 Sofia, Bulgaria; 2 Department of Organisation and Economics of Pharmacy, Faculty of Pharmacy, Medical University of Plovdiv, 4002 Plovdiv, Bulgaria; radiana.staynova@mu-plovdiv.bg; 3 Department of Health Economics, Faculty of Public Health “Prof. Tsekomir Vodenicharov, MD, DSc”, Medical University of Sofia, 1527 Sofia, Bulgaria; e.naseva@foz.mu-sofia.bg

**Keywords:** pregnancy, trimester specific, thyroid-stimulating hormone, reference interval, Bulgaria

## Abstract

*Background and Objectives*: Pregnancy introduces various interfering factors that, alongside individual variations, impact the assessment of thyroid function tests. This underscores the necessity of defining trimester-specific reference intervals for thyroid-stimulating hormone (TSH) levels. Differences in population characteristics, including ethnicity, socio-economic factors, iodine prophylaxis, and obesity, emphasize the need to establish trimester-specific TSH ranges for women of reproductive age in the respective region or center. The aim of the present study was to establish first- and second-trimester-specific reference intervals for TSH and free thyroxine (FT4) in a relevant pregnant population. *Materials and Methods*: A retrospective monocenter analysis utilized the electronic database of Ob/Gyn Hospital “Dr. Shterev”, Sofia, Bulgaria. The analysis involved data from 497 pregnant and 250 non-pregnant women, all without evidence of thyroid dysfunction or a family history thereof, no indication of taking medication interfering with thyroid function, no evidence of levothyroxine treatment, and no history of sterility treatment. To establish the limits of the TSH reference range, the percentile method was applied using a bootstrapping procedure following the recommendations of the International Federation of Clinical Chemistry (IFCC). *Results*: Trimester-specific reference intervals for TSH and FT4 in our center were established as follows: first trimester—0.38–2.91 mU/L, FT4-12.18–19.48 pmol/L; second trimester—0.72–4.22 mIU/L and 9.64–17.39 pmol/L, respectively. We also established the normal reference range for the non-pregnant control group, which is similar to that applicable in our laboratory. *Conclusions*: Our results differ from the fixed limits recommended by the American Thyroid Association, European Thyroid Association, and Endocrine Society Guidelines. Following the relevant established intervals would significantly impact timely diagnosis and therapy requirements for a substantial proportion of pregnant women.

## 1. Introduction

Pregnancy is associated with the presence of interfering factors such as levels of estrogens, thyroxine-binding globulin, human chorionic gonadotropin, iodine sufficiency, and deiodinase activity, as well as the presence of antithyroid antibodies [1]. These factors, alongside individual variations in pregnant women, can impact the assessment of thyroid hormone levels. Consequently, general population ranges for thyroid-stimulating hormone (TSH) levels do not apply to pregnant women, and it is recommended to use trimester-specific reference intervals [2,3,4,5,6]. The adoption of such intervals significantly alters the approach to assessing thyroid dysfunction in pregnant women and initiating related therapies [2,7]. The importance of using trimester-specific intervals comes from the fact that even mild fluctuations in thyroid function could be associated with complications during pregnancy [8,9].

In many countries, including Bulgaria, established reference ranges for TSH during pregnancy are lacking [2,10,11,12]. Consequently, recommendations and criteria from major professional associations are relied upon [3,4,5,6]. Population studies have informed opinions from the American Thyroid Association (ATA), European Thyroid Association (ETA), and Endocrine Society, suggesting fixed levels for thyroid hormones. While standardizing diagnostic criteria is advantageous, strict adherence to fixed TSH limits during pregnancy may lead to unnecessary diagnosis and treatment, potentially causing complications [2]. Variations between populations in ethnicity, socio-economic factors, iodine prophylaxis, and obesity emphasize the need for trimester-specific ranges for thyroid hormone levels in women of reproductive age, specific to each region or center [2,13,14]. International guidelines suggest employing fixed ranges for thyroid hormones in centers unable to calculate such intervals [3,4,5,6]. According to guidelines published by ETA, ATA, and the Endocrine Society, the upper reference interval for TSH during the first trimester remained at 2.5 mIU/L for an extended period [3,4,5]. However, many studies published after 2011 indicate that this limit increases the diagnosis of subclinical hypothyroidism, leading to overdiagnosis and unnecessary treatment [11,12,15,16,17,18,19]. Furthermore, TSH levels associated with FT4 reduction typically fall between 4 and 5 mIU/L. Consequently, in 2017, ATA revised its recommendations, setting the upper reference range for TSH in women without autoimmune thyroid pathology at 4 mIU/L, while maintaining the interval of 0.3–2.5 mIU/L for patients with thyroid autoimmunity [3].

Several factors contribute to determining trimester-specific reference intervals, including race, maternal age, gestational age, presence of antibodies, iodine sufficiency, parity, multiple pregnancies, sampling time, body mass index, and smoking [20].

According to the International Federation of Clinical Chemistry recommendations for thyroid diagnosis and therapy, the reference interval should span from the 2.5th to the 97.5th percentile of individuals with normal iodine intake and no evidence of autoimmune thyroid diseases [16]. Some authors suggest expressing TSH and FT4 levels as a multiple of the median (MoM), derived by dividing each value by the population mean. This standardized approach ensures independence from measurement-related variables. Establishing a reliable reference interval requires a minimum of 120 patients [16].

The aim of the present study was to establish first- and second-trimester-specific reference intervals for TSH and free thyroxine (FT4) in a relevant pregnant population.

## 2. Materials and Methods

### 2.1. Study Design and Setting

A monocentric retrospective analysis was conducted using the electronic database of “Dr. Shterev” Hospital, Sofia, Bulgaria. The study followed national and international ethical requirements, ensuring participant anonymity and confidentiality of personal information. Ethical approval was received from the Institutional Review Board of the Specialized Hospital for Obstetrics and Gynecology “Dr. Shterev”, Sofia, Bulgaria (Protocol No. 466/2018).

### 2.2. Study Population

**Figure 1:**
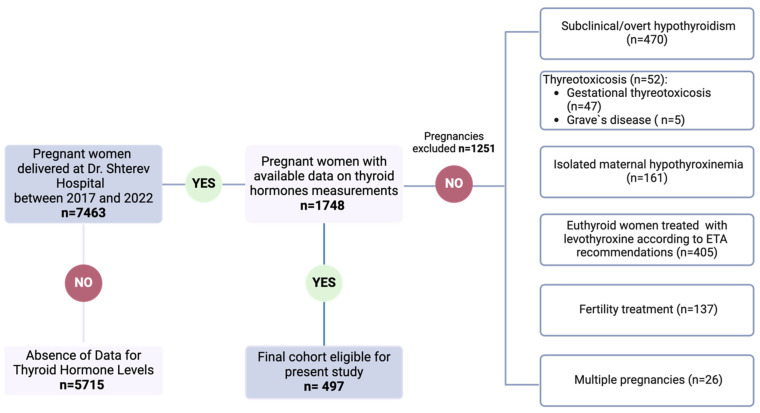
Flowchart for pregnant cohort selection (created with BioRender.com, accessed on 8 March 2024).

Data from 7463 Caucasian patients who delivered at our hospital between 2017 and 2022 were analyzed. Among this cohort, 497 patients met the inclusion criteria and were included in the analysis (Figure 1). Although patients originated from different regions across the country, the majority (63%) hailed from the capital city.

#### 2.2.1. Inclusion Criteria

- Pregnant women with documented TSH and FT4 levels at the time of pregnancy detection; spontaneous, singleton pregnancy;
- Maternal age between 18 and 40 years;
- Absence of thyroid dysfunction (e.g., goiter, cancer, hyperthyroidism, hypothyroidism) and/or autoimmunity;
- No family history of thyroid dysfunction;
- No clinical diagnosis of diabetes mellitus;
- No history of past or current intake of medications affecting thyroid function (levothyroxine, propylthiouracil, methimazole, glucocorticoids, etc.)

#### 2.2.2. Exclusion Criteria

Euthyroid pregnant women with positive anti-TPO and/or anti-Tg antibodies, those conceiving via assisted reproductive technologies, individuals with established thyroid pathologies, those taking levothyroxine or other thyroid-affecting medications, those with multiple pregnancies, and those with a family history of thyroid pathology were excluded from the analysis.

Thyroid hormone tests were conducted during the first trimester (up to 12 weeks gestation) in all 497 patients and the second trimester (up to 24 weeks gestation) in 367 of these pregnant women. Gestational age was determined based on the last menstrual period and ultrasound examination. Antithyroid antibodies were screened once during the first trimester of pregnancy, parallel to the initial thyroid hormone assessment.

A control group consisting of 250 healthy, non-pregnant women of reproductive age was chosen to facilitate the comparison of TSH and FT4 levels between pregnant and non-pregnant cohorts. The control group was also recruited through the electronic database of the “Dr. Shterev” Hospital, Sofia, Bulgaria. We selected non-pregnant women without clinical and laboratory evidence of thyroid dysfunction, autoimmune thyroid disease, family history of thyroid disease, or medication use affecting thyroid function. The two groups were paired and did not differ significantly in terms of age and body mass index. The comparison aimed to elucidate the disparity in thyroid hormone levels between matched healthy pregnant and non-pregnant populations.

Urine iodine testing was not conducted; however, Bulgaria is classified as an iodine-sufficient country. This classification is supported by a 2020 population study by Anna-Maria Borisova et al., demonstrating iodine sufficiency among the studied population of pregnant Bulgarian women [21].

### 2.3. Laboratory Analysis

TSH levels were determined using the immuno-chemiluminescence method (Roche Cobas 8000, Basel, Switzerland), with the laboratory’s reference interval being 0.27–4.20 mUI/L. FT4 levels were measured using the same method, with the laboratory’s reference interval being 12–22 pmol/L. Antibody levels were assessed using the electro-chemiluminescence method (Roche Cobas 8000, Switzerland). The interassay coefficients of variation were 1.8% for TSH, 1.6% for FT4, and <13% for antithyroid antibodies. The lower limit of detection for TSH was 0.005 mIU/L. Ultrasound data for the thyroid gland were available for all patients included in the analysis, and no structural changes were observed.

### 2.4. Statistical Analysis

All data were analyzed using SPSS software version 24.0 (SPSS, Inc., Chicago, IL, USA). Results are presented as medians and interquartile ranges (IQR: 25th and 75th percentiles) due to their non-Gaussian distribution. The distribution shape was assessed using the Kolmogorov–Smirnov test. Average values between the two groups were compared using the Mann–Whitney U test, while paired samples were compared using the Wilcoxon test. A *p*-values less than 0.05 was considered statistically significant. Bootstrapping was performed for 1000 samples, and 95% confidence intervals were calculated.

## 3. Results

Our cohort comprised 7463 pregnancies, of which 1748 had available data on thyroid hormone measurements. Among them, 1251 were excluded from the analysis due to not meeting the inclusion criteria. The final cohort eligible for the current study consisted of 497 pregnant women (Figure 1).

The main characteristics and thyroid hormone levels of the study participants are shown in Table 1. The median age of pregnant women and controls was 31 years (IQR 29–34 years) and 32 years (IQR 29–35 years), respectively. There were no significant differences between pregnant and non-pregnant women regarding age and BMI. The median gestational ages for the first and second trimesters were 12.4 weeks (range: 6.6–13.1) and 16.1 weeks (range: 13.6–23.6), respectively. Thyroid function tests were conducted during the first trimester (up to 12 weeks gestation) in all 497 patients and during the second trimester (up to 24 weeks gestation) in 367 of these pregnant women.

The median TSH and FT4 levels of pregnant women were compared with those of 250 non-pregnant women. Serum TSH levels in healthy pregnant women were significantly lower than those in healthy non-pregnant women (*p* < 0.001). We observed statistically significant differences between trimesters for TSH levels (*p* < 0.001) and also for FT4 (*p* < 0.001). According to our results, the median TSH levels during the second trimester were significantly higher, while median FT4 levels were significantly lower compared with those of the first trimester (Table 1).

Based on the established recommendations of the International Federation of Clinical Chemistry (IFCC), reference intervals for TSH and FT4 were determined using the percentiles method with a bootstrapping procedure. Results are presented as medians, lower and upper limits of the reference intervals (2.5 and 97.5 percentiles), provided with 95% confidence intervals. Trimester-specific reference intervals for thyroid hormones (TSH and FT4) are outlined in Table 2 and Table 3.

Following the analysis and adhering to the methodological requirements for establishing reference intervals, the determined limits of TSH levels for the first and second trimesters in our medical center were 0.49–2.91 mIU/L and 0.73–4.22 mIU/L, respectively (Table 2). Reference ranges for FT4 values were 12.18–19.48 pmol/L and 9.46–17.40 pmol/L for the first and second trimesters, respectively (Table 3). Furthermore, we determined the normal reference range for the non-pregnant control group, which aligns with the standards applicable in our laboratory (Table 2 and Table 3)

## 4. Discussion

In Bulgaria, there is a lack of population-based studies aimed at establishing trimester-specific reference intervals for TSH levels in pregnant women. To address this gap, the Bulgarian Society of Endocrinology [10] has adopted the recommendations of the Endocrine Society, which propose fixed reference intervals for TSH levels in the absence of trimester-specific data. These fixed intervals are as follows: first trimester: 0.1–2.5 mIU/L; second trimester: 0.2–3.0 mIU/L; third trimester: 0.3–3.0 mIU/L [4].

Numerous studies published since 2011 have indicated that the upper limit of TSH recommended by these guidelines may lead to an increased diagnosis of subclinical hypothyroidism, resulting in overdiagnosis and unnecessary treatment in a significant proportion of patients. Studies conducted in various populations worldwide have reported that between 8 and 28% of pregnant women without complications have TSH levels exceeding the generally accepted cutoff of 2.5 mIU/L [21,22,23,24,25].

Our study aims to compare TSH levels during pregnancy, measured under standardized conditions at our clinical center, with the established trimester-specific laboratory ranges widely accepted for all pregnant women. Through this comparison and considering the characteristics of our study population, we seek to determine whether our findings and analyses can serve as a representative reference for diagnosing thyroid dysfunction in pregnant women. This approach will enable the evaluation of the applicability of the generally accepted TSH reference interval as a diagnostic tool in our specific pregnant population.

The trimester-specific reference ranges for TSH that we established diverge from the fixed intervals recommended by the ATA and the Endocrine Society [3,4].

Our findings demonstrate variations in TSH and FT4 levels across different trimesters in pregnant women compared to those in non-pregnant women. Consistent with previous research, our study confirms a trend of increasing TSH and decreasing FT4 levels from the first to subsequent trimesters, contrasting with levels observed in non-pregnant individuals.

These results align with findings from studies conducted in pregnant populations of diverse ethnicities and environmental contexts regarding iodine sufficiency, where deviations from commonly accepted fixed reference ranges for TSH were noted. Recent investigations worldwide focusing on trimester-specific TSH reference intervals have consistently reported discrepancies from widely used fixed limits. Studies conducted across various regions of China, for instance, demonstrated variations not only from established guidelines but also among different provinces [26,27,28,29].

For example, a study conducted by Sheng et al. in Guangxi Province, China, reported trimester-specific TSH reference intervals of 0.02–0.32 for the first trimester and 0.03–3.22 mIU/L for the second trimester [30]. Similarly, research conducted in Beijing, China, indicated reference intervals of 0.59–3.4 for the first trimester and 0.8–4.46 mIU/L for the second trimester [18]. These findings underscore the influence of environmental and population-specific factors on thyroid function test results, even within a single country.

The established trimester-specific reference intervals differ from the fixed and established ones, which would significantly change the strategy for initiating pharmacotherapy in pregnant women. Comparable deviations from established TSH reference limits in pregnancy have been documented in studies from various countries, including Japan, Turkey, UAE, India, Korea, Brazil, Poland, Pakistan, and Chile [2,11,19,22,23,31,32,33,34]. For instance, a study by Morais et al. identified an upper limit for TSH in postnatal pregnant women corresponding to fixed reference ranges recommended by the ATA [24].

The variations in our trimester-specific reference ranges, alongside disparities noted in published data from international studies, prompt questioning the accuracy of thyroid function test interpretation in pregnant women. Following fixed reference ranges often results in unnecessary or untimely treatment, posing risks of both delayed diagnosis and treatment and overdiagnosis, which can lead to complications during pregnancy [9,35,36,37,38].

Our study has several limitations. Firstly, its retrospective design and lack of information regarding the timing of thyroid function testing pose constraints. Additionally, the inability to establish TSH and FT4 reference ranges for the third trimester represents another limitation. The number of patients in whom thyroid hormone levels were assessed in the third trimester and who met the inclusion criteria for establishing a reference interval did not adequately correspond to the recommended sample size. Consequently, reference ranges for TSH during the third trimester were not established. Given that FT4 levels during pregnancy are influenced by estrogens, thyroxin-binding globulin, and albumin, mitigating these confounding factors is crucial for determining trimester-specific FT4 reference intervals through specific analyses and methodologies. Another limitation to be acknowledged was the lack of data for urine iodine testing. Although urine iodine testing was not conducted, Bulgaria is classified as an iodine-sufficient country The data regarding iodine sufficiency can be accepted as completely reliable, following the results of a population study by Anna-Maria Borisova et al., which demonstrated iodine sufficiency among a studied cohort of pregnant women [21].

## 5. Conclusions

Diagnosing thyroid dysfunction during pregnancy presents a significant challenge, necessitating a comprehensive understanding of the various factors influencing maternal thyroid function. Using trimester-specific intervals is imperative because even minor fluctuations in thyroid function can contribute to pregnancy complications. However, reliance on fixed reference ranges often prompts unnecessary therapeutic interventions, compromising individualized care and treatment adequacy. Adhering to the appropriate trimester-specific intervals would markedly alter the diagnosis and therapeutic requirements for a substantial proportion of pregnant patients in Bulgaria.

## Figures and Tables

**Table 1:** Main characteristics and thyroid hormone levels of study participants.

Variables	Pregnant Women (*n* = 497, 1st Trimester *n* = 367, 2nd Trimester)	Non-Pregnant Women (*n* = 250)	*p*-Value
Age (years) *	31 (29–34)	32 (29–35)	0.402
Gestational age (weeks)		-	-
1st trimester **	12.4 (6.6–13.1)		
2nd trimester **	16.1 (13.6–23.6)		
BMI (kg/m^2^) *	23 (20–26)	24 (22–26)	0.503
TSH (mIU/L)		2.50 (1.62–3.60)	0.001 ^a,b^
1st trimester *	1.70 (1.30–2.19)		
2nd trimester *	2.46 (2.03–2.98)		
FT4 (pmol/L)		13.80 (12.54–15.60)	0.001 ^a,b^
1st trimester *	15.08 (13.50–16.60)		
2nd trimester *	13.20 (12.03–14.76)		

* Data are presented as median (IQR) for age, BMI, TSH, and FT4; ** Data are presented as median (min–max) for gestational age. ^a^ pregnant vs. non-pregnant women; ^b^ 1st vs. 2nd trimester. Abbreviations: BMI, Body Mass Index; TSH, Thyroid-stimulating hormone; FT4, Free thyroxin.

**Table 2:** Established trimester-specific reference intervals for TSH.

Groups		TSH (mIU/L)		
	N	Median	Percentiles	
			2.5th (95% CI)	97.5th (95% CI)
**Non-pregnant women**	250	2.49	0.41 (0.25–0.55)	4.53 (4.23–5.51)
**Pregnant women**				
1st trimester	497	1.70	0.49 (0.30–0.61)	2.91 (2.76–3.12)
2nd trimester	367	2.46	0.73 (0.39–0.89)	4.22 (4.02–4.98)

Abbreviations: TSH, Thyroid-stimulating hormone; FT4, Free thyroxin; CI, Confidence Interval.

**Table 3:** Established trimester-specific reference intervals for FT4.

Groups		FT4 (pmol/L)		
	N	Median	Percentiles	
			2.5th (95% CI)	97.5th (95% CI)
**Non-pregnant women**	250	13.80	9.65 (9.25–11.20)	19.80 (18.47–19.80)
**Pregnant women**				
1st trimester	497	15.08	12.18 (12.04–12.20)	19.48 (19.00–19.77)
2nd trimester	367	13.20	9.46 (9.03–10.30)	17.40 (16.80–18.07)

Abbreviations: FT4, Free thyroxin; CI, Confidence Interval.

## Data Availability

All data presented in this study are available from the corresponding author upon reasonable request.
